# Comparative testicular transcriptomic analysis reveals hub genes and pathways regulating semen quality in Zhedong white ganders (*Anser cygnoides*)

**DOI:** 10.3389/fvets.2026.1906421

**Published:** 2026-08-12

**Authors:** Lei Zhang, Guobo Sun, Rui Zhu, Xiaoming Li, Gansheng Zhang, Xiujun Duan

**Affiliations:** 1 School of Modern Animal Husbandry, Jiangsu Agri-animal Husbandry Vocational College, Taizhou, China; 2 Jiangsu Key Laboratory for High-Tech Research and Development of Veterinary Biopharmaceuticals, Engineering Technology Research Center for Modern Animal Science and Novel Veterinary Pharmaceutic Development, Taizhou, China

**Keywords:** goose, reproductive physiology, RNA-Seq, semen quality, testis

## Abstract

**Introduction:**

Semen quality is a critical determinant of reproductive efficiency in poultry, yet its molecular regulation remains poorly understood.

**Methods:**

To identify key genes associated with semen quality in ganders, individuals were stratified into high (H) and low (L) semen quality groups based on a composite sperm quality factor (SQF). Cross-group comparisons of semen parameters, testicular histomorphology, and external genitalia morphology were conducted, followed by high-throughput transcriptomic analysis of testicular tissue.

**Results:**

The H group exhibited significantly superior semen quality, testicular development, and external genitalia morphology compared to the L group (P < 0.01). Transcriptomic profiling identified 3,334 differentially expressed genes (DEGs), with 1,552 upregulated and 1,782 downregulated in the H group. GO enrichment analysis revealed that DEGs were primarily involved in biological processes related to cell adhesion, cell differentiation, steroid biosynthetic process, and calcium ion binding. KEGG pathway enrichment analysis further identified ECM-receptor interaction, calcium signaling, and Wnt signaling pathways as potential key regulatory pathways underlying semen quality differences between groups. Protein–protein interaction (PPI) network analysis further identified 11 hub genes (*COL1A1*, *HGF*, *COL1A2*, *ITGA5*, *NGFR*, *TWIST1*, *COL6A2*, *DKK1*, *BMP7*, *WNT2*, and *CYP11A1*) with potential roles in spermatogenesis.

**Conclusion:**

These findings elucidate the molecular basis for semen quality differences in ganders, advance avian reproductive biology research, and identify potential selective breeding markers to enhance commercial goose production efficiency.

## 1 Introduction

The domestic goose (*Anser cygnoides*) is an economically vital waterfowl in modern animal husbandry, with commercial breeding enterprises widely established across major agricultural regions in the world (1). According to FAO statistical data (2), China dominates global goose production and consumption, contributing more than 95% of the world’s total goose meat production. Goose-derived products, including meat, eggs, liver, and feathers, possess substantial market potential and economic value (3). Compared with chickens and ducks, geese exhibit generally inferior reproductive performance in terms of fertility and hatchability, which substantially limits the development of global goose farming (4). Such low reproductive efficiency is primarily attributed to two major constraints: low annual egg production in female geese, poor semen quality in male geese, both of which severely impede large-scale commercial breeding applications (5, 6). This collectively indicates that suboptimal reproductive performance in geese arises from dual limitations across both sexes. Numerous research groups have conducted studies on the reproductive performance of female geese, including genetic regulation (7), endocrine mechanisms (8), seasonality (9), broody behavior (10), and so on. However, compared with the extensive research on female goose reproductive performance, studies on male goose reproductive performance, particularly semen quality, remain relatively limited.

Semen quality is a key factor for fertilization success in goose breeding. It is also a highly heritable quantitative trait in poultry, largely controlled by genetic factors (11). For example, the heritability of sperm motility in Beijing-You chickens reaches as high as 0.85 (4). A previous study showed that slow-feathering Royal Princess roosters had significantly higher ejaculate volume, sperm density, and sperm motility than fast-feathering ones (12). Łukaszewicz et al. (6) compared semen quality across six goose breeds. They found that Kartuska ganders had the highest sperm motility (53.8%), whereas Kielecka ganders showed the lowest (43.6%). In recent years, more functional genes related to sperm motility have been identified in poultry. Imsland et al. (13) confirmed that disruption of the *CCDC108* transcript contributes to reduced sperm motility in rose-comb roosters. In 2023, Lukaszewiczl et al. (14) proposed the sperm quality factor (SQF), a composite metric integrating ejaculate volume, sperm concentration and the percentage of live normal spermatozoa. SQF is a reliable semen quality indicator and has been successfully integrated into routine reproductive assessment protocols by leading experts in animal husbandry (15). Using this indicator, Liu et al. (16) documented a broad rage in SQF (14.32–146.83) of Zhedong white goose, highlighting the critical importance of implementing rigorous selection protocols for superior males to enhance fertilization efficiency and reproductive performance in commercial goose operations. Hu et al. (17) compared the SQF of Tianfu Meat Goose II across three stages of female geese’s annual laying cycle and explored significantly greater testicular weight and size in the high SQF group than in the low SQF group (*p* < 0.05). Moreover, testicular RNA-Seq analysis suggested that the hub genes *EP300*, *RAB31*, *PRKG1*, and *PIK3R1* may be associated with goose semen quality.

The overall reproductive capacity of ganders is largely determined by semen quality, which directly governs fertilization efficiency in breeding practice. Therefore, identifying key molecular markers and regulatory pathways that control gander semen quality is essential for improving male reproductive performance. Despite considerable advancements in understanding avian reproductive physiology, the intricate molecular pathways orchestrating testicular morphogenetic programming, reproductive functional homeostasis, and semen quality trait modulation in ganders remain largely uncharacterized. In the present study, we divided ganders into high- and low-semen-quality groups based on SQF. We then compared testicular and external genital morphological and histological differences between the two groups. Additionally, we performed testicular transcriptome sequencing to screen hub genes and core signaling pathways regulating gander semen quality. Our findings aim to reveal the molecular regulatory mechanisms underlying gander reproductive performance, provide valuable genetic resources for molecular breeding of high-fertility ganders, promote goose germplasm innovation, and support the sustainable development of the modern goose industry.

## 2 Materials and methods

### 2.1 Ethics statement

This study was approved by the Institutional Animal Ethics Committee of Jiangsu Agri-animal husbandry vocational college, Taizhou, Jiangsu, China (Approval No: jsahvc-2023-95). All experimental procedures were carried out in accordance with the guidelines for the care and utility of experimental animals by the Ministry of Agriculture of China.

### 2.2 Experimental animals, semen quality and sampling

A total of 60 healthy 160-day-old male Zhedong geese were reared in individual cages under the same environmental and feeding conditions. All ganders were subjected to a one-month adaptation period in individual cages prior to semen collection to minimize stress and ensure the success of subsequent artificial semen collection and training procedures. Following this adaptation period, all ganders underwent a 7-day training period for semen collection using the massage method. Following training, semen was collected from each gander once every 3 days, for a total of five collections. Finally, valid semen data were obtained from 34 ganders. The collected semen at each sampling time was subjected to semen quality analysis, including the ejaculate volume, sperm density, sperm motility, sperm deformity rate were measured and the SQF value of each gander was calculated as described (18). Based on individual SQF, the ganders were divided into two groups: the low sperm quality (**L**) group and the high sperm quality (**H**) group. Given that semen quality from artificial collection can be influenced by multiple factors, the five ejaculates collected from each individual exhibited considerable variability. To ensure within-group consistency, individuals with SQF standard deviation values below 5 in the L group and below 40 in the H group were further included.

After semen collection, six ganders were randomly selected from the above two groups and subjected to a 12-h fast prior to live body weight measurement. Euthanasia was performed by carbon dioxide inhalation followed by exsanguination via cervical dislocation. After euthanasia, the external genitals of each gander were examined first and then the blood samples were collected from each gander. The both the left and right testes were weighed, measured and photographed. A tissue segment from left-side testis was collected and promptly snap-frozen in liquid nitrogen for subsequent experiments, while the contralateral testis was entirely fixed in 4% formaldehyde for subsequent paraffin sectioning.

### 2.3 Morphological and histological observation

The external genitals were observed and parameters including number of helices, basal diameter, natural length and stretched length were recorded. The testes were observed and measured with long, short, and dorsoventral diameters of both left and right testis. The reproductive organ index was calculated using the following formula: reproductive organ index (%) = [reproductive organ weight (g)/ body weight (kg)] × 100%.

The collected testes were made to 5 μm thick H&E-stained slices, then observed and photographed under Pannoramic MIDI microscope slide scanner (3DHISTECN, Hungary, Budapest). Then the Image-Pro Plus 6.0 software (National Institutes of Health, Bethesda, MD, USA) was used to measure the SET (seminiferous epithelial thickness), STD (seminiferous tubule diameter), TI (testicular interstitium) and TP (testicular parenchyma).

### 2.4 Testes transcriptome sequencing and data analysis

Total RNA was extracted, qualified and transported to OE biotech Co., Ltd. (Shanghai, China) for transcriptome sequencing (RNA-Seq). The libraries were sequenced on an Illumina HiSeq 2,500 system sequencing platform. The clean reads were mapped to the Taihu goose T2T genome (GCF_040182565.1) using hisat2 (2.0.5) for the downstream analysis as previously described (19). The differential expressed genes (DEGs) between samples were identified using the DESeq2 R package (1.20.0) (20), and *q* value < 0.05 and |log_2_foldchange| ≥ 1 were used as the criteria of significance of DEGs. GO term and KEGG pathway analyses were performed by clusterProfiler software (4.0) (21). The STRING database (22) was used to explore the interaction between DEGs. The raw sequence data reported in this paper have been deposited in the Genome Sequence Archive (23) in National Genomics Data Center (24), China National Center for Bioinformation/Beijing Institute of Genomics, Chinese Academy of Sciences (GSA: CRA030664) that are publicly accessible at https://ngdc.cncb.ac.cn/gsa/s/fIl9N5BK.

### 2.5 Quantitative reverse transcription polymerase chain reaction (qRT-PCR) analysis

To validate the RNA-Seq results, the expression patterns of 6 randomly selected DEGs was assessed using qRT-PCR method. Equal amounts of total RNA were reversely transcribed into the cDNAs using the SuperScript III First-Strand Synthesis SuperMix (Thermo Fisher, Nanjing, China). The primers (Supplementary Table 1) were designed using Primer 5.0 software. qPCR was performed on an CFX96 Real-Time System (Bio-Rad, Hercules, CA, USA). Each reaction was performed in a 20 μL volume, containing 10 μL 2 × ChamQ SYBR qPCR Master Mix (Vazyme, Nanjing, China), 0.4 μL of each forward and reverse primer, 2 μL cDNA, 7.2 μL nuclease-free water. The cycling protocol included an initial 30 s denaturation at 95 °C, followed by 40 cycles of 5 s at 95 °C and 30 s at the optimal annealing temperature. To ensure assay reliability, both no-template controls and negative controls without reverse transcriptase were also included in each run. Target specificity for each primer set was validated by melting curve analysis. Each sample was run in triplicate. The relative mRNA expression levels of these DEGs were normalized using the two reference genes *GAPDH* and *β-ACTIN* according to the comparative Ct method (18).

### 2.6 Statistical analysis

All statistical analyses in this study were performed using SPSS, version 19.0 (IBM, Armonk, NY, USA). The results were expressed as mean ± standard deviation. Differences were assessed using the independent-sample *t*-test. A *p-value* < 0.05 was statistically significant, and *p-value* < 0.01 was highly statistically significant.

## 3 Results

### 3.1 Comparison of semen quality of ganders between H and L group

**Figure 1:**
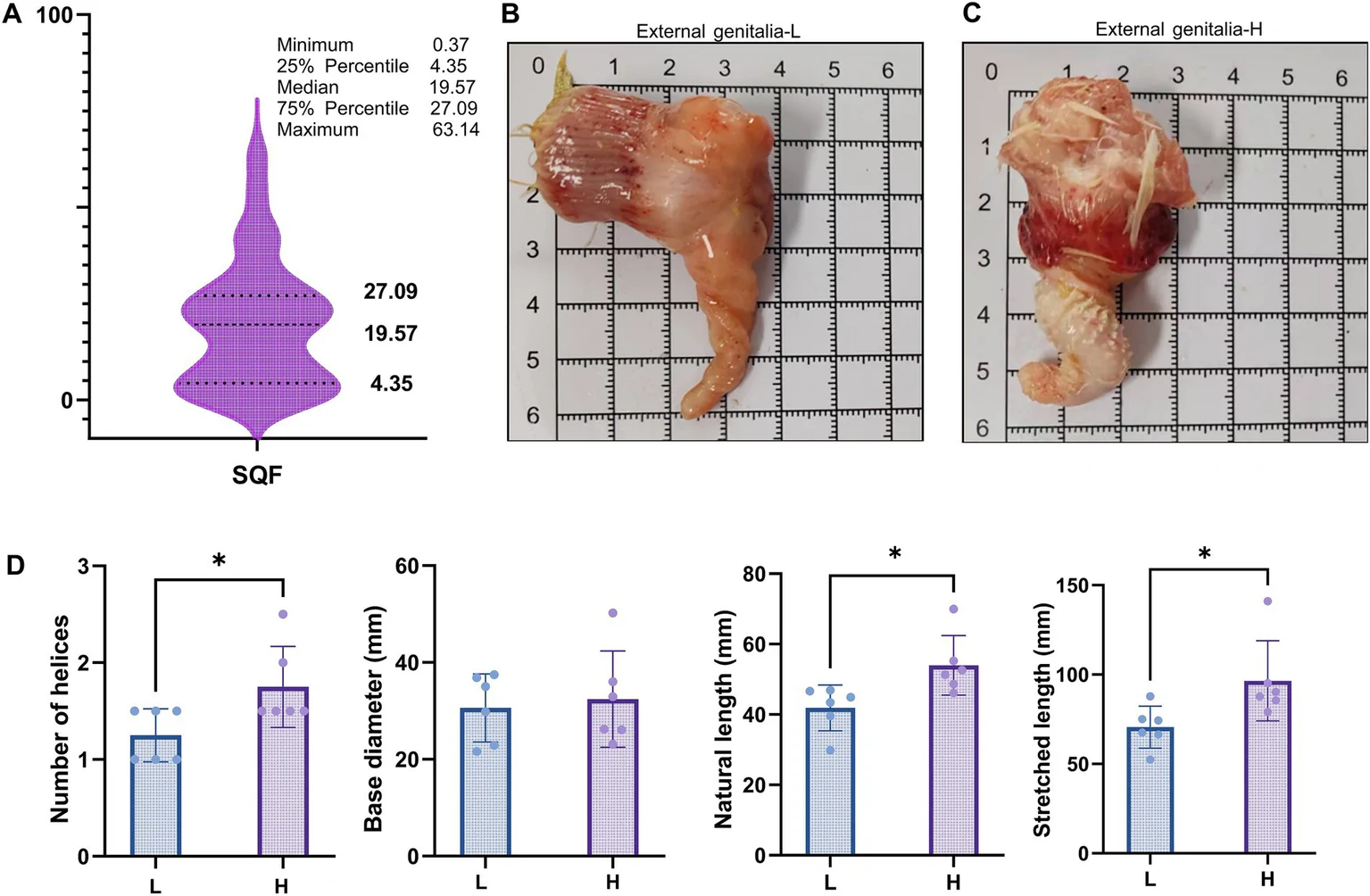
Total SQF distribution and external genitalia comparison between L and H groups. **(A)** Distribution of SQF among 34 ganders. **(B)** Representative external genitalia morphological observations of ganders from the L group. **(C)** Representative external genitalia morphological observations of ganders from the H group. **(D)** External genitalia parameters comparisons of ganders between the two groups, * indicates a significant difference (*p* < 0.05).

As shown in Figure 1A, the median SQF of all ejaculated ganders was 19.57, with the top and lower quartile were 27.09 and 4.35, respectively. Accordingly, the ganders with SQF above 27.09 were classified in to H group, while those below 4.35 were classified into the L group. Subsequently, six ganders from each group were selected to compare semen quality. As shown in Table 1, the ejaculate volume, sperm density, sperm motility, and SQF of ganders were highly statistically significant higher in H group than in L group (*p* < 0.01), while the sperm deformity rate was highly statistically significant lower in H group than in L group (*p* < 0.01).

**Table 1:** Comparison of the semen quality from ganders between L and H groups.

Indicator	L group (*n* = 6)	H group (*n* = 6)	*p* value
Ejaculate volume (mL)	0.44 ± 0.10	0.64 ± 0.15	0.0009
Sperm density (10^8^/mL)	0.34 ± 0.23	1.63 ± 0.70	<0.0001
Sperm motility (%)	20.55 ± 15.62	39.89 ± 8.97	0.0007
Sperm deformity rate (%)	35.93 ± 19.16	15.00 ± 2.99	0.0001
SQF	1.78 ± 1.71	35.15 ± 25.66	0.0022

### 3.2 Morphological and histological differences between H and L group

**Figure 2:**
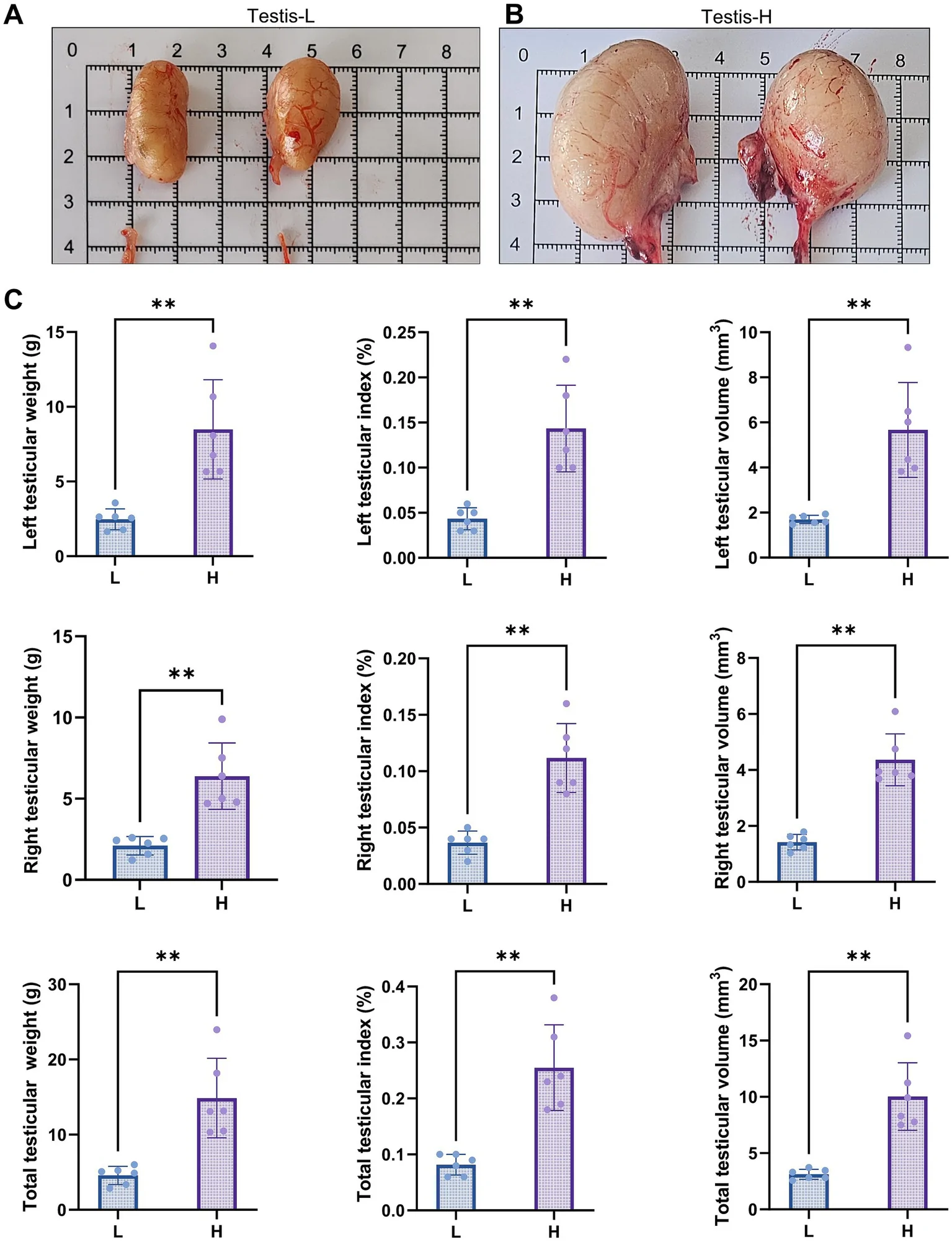
Comparison of the testicular morphology between L and H groups. **(A)** Testicular histological section from the L group. **(B)** Testis histological section from the H group. **(C)** Comparison of histological parameters (including seminiferous tubule diameter and epithelial height) between the two groups, * indicates a significant difference (*p* < 0.05), ** indicates an extremely significant difference (*p* < 0.01).

**Figure 3:**
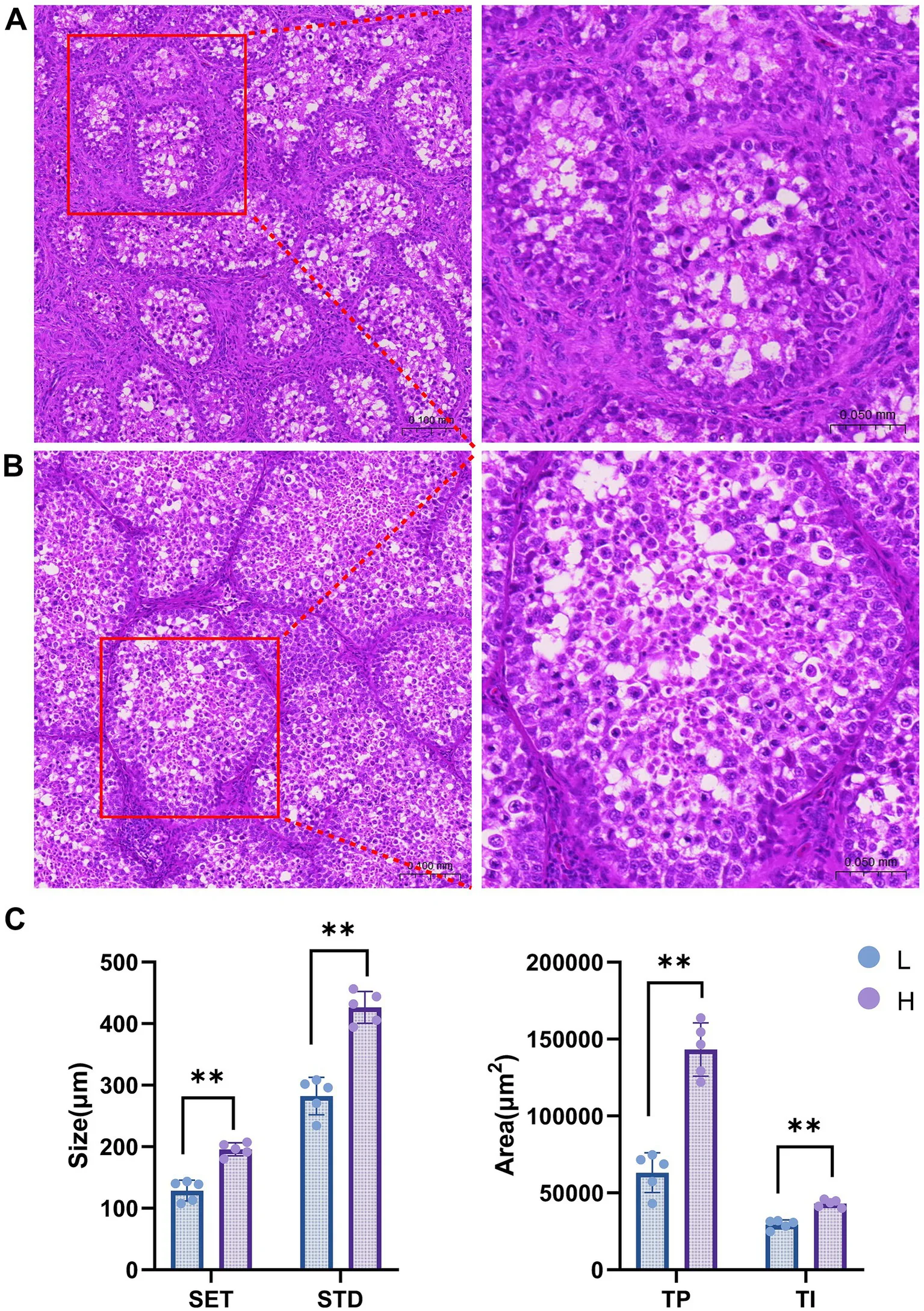
Comparison of the testicular morphology between L and H groups. **(A)** Testicular morphological observations of ganders from the L group. **(B)** Testicular morphological observations of ganders from the H group. **(C)** Comparison of testicular histological parameters between the two groups, ** indicates an extremely significant difference (*p* < 0.01).

External genital morphology was compared between the two groups (Figures 1B,C). Compared with the L group, the H group exhibited significantly increased helical number, natural length, and stretched length of the external genitalia (*p* < 0.05), with no significant difference in basal diameter (Figure 1D). Meanwhile, testicular morphology between L and H groups was also compared between the two groups (Figures 2A,B). All measured testicular parameters, including weight, index, and volume of the left, right, and total testes, were significantly higher in the H group than the L group (*p* < 0.01, Figure 2C). At the same magnification, Figure 3A (Group L) showed a greater number of seminiferous tubules per unit area, but with smaller diameters, sparsely arranged and inconsistent in size, accompanied by increased interstitial tissue. Under high magnification, the seminiferous epithelium exhibited few cell layers, containing only occasional early-stage germ cells and Sertoli cells, with only a limited number of mature spermatozoa. In contrast, Figure 3B (Group H) showed fewer seminiferous tubules per unit area, but with larger diameters, densely packed and regular in size, with moderate interstitial tissue, and multiple layers of spermatogenic cells at various developmental stages arranged in an orderly manner, with abundant mature spermatozoa. These observations reveal marked differences in testicular tissue structure and spermatogenic development between the two groups. Furthermore, statistical analysis (Figure 3C) demonstrated that, the size of SET and STD in group H were significantly higher than those in group L (*p* < 0.01). In addition, compared with group H, group L had a significantly smaller TP and TI area (*p* < 0.01).

### 3.3 Identification and functional analysis of differentially expressed genes (DEGs)

**Figure 4:**
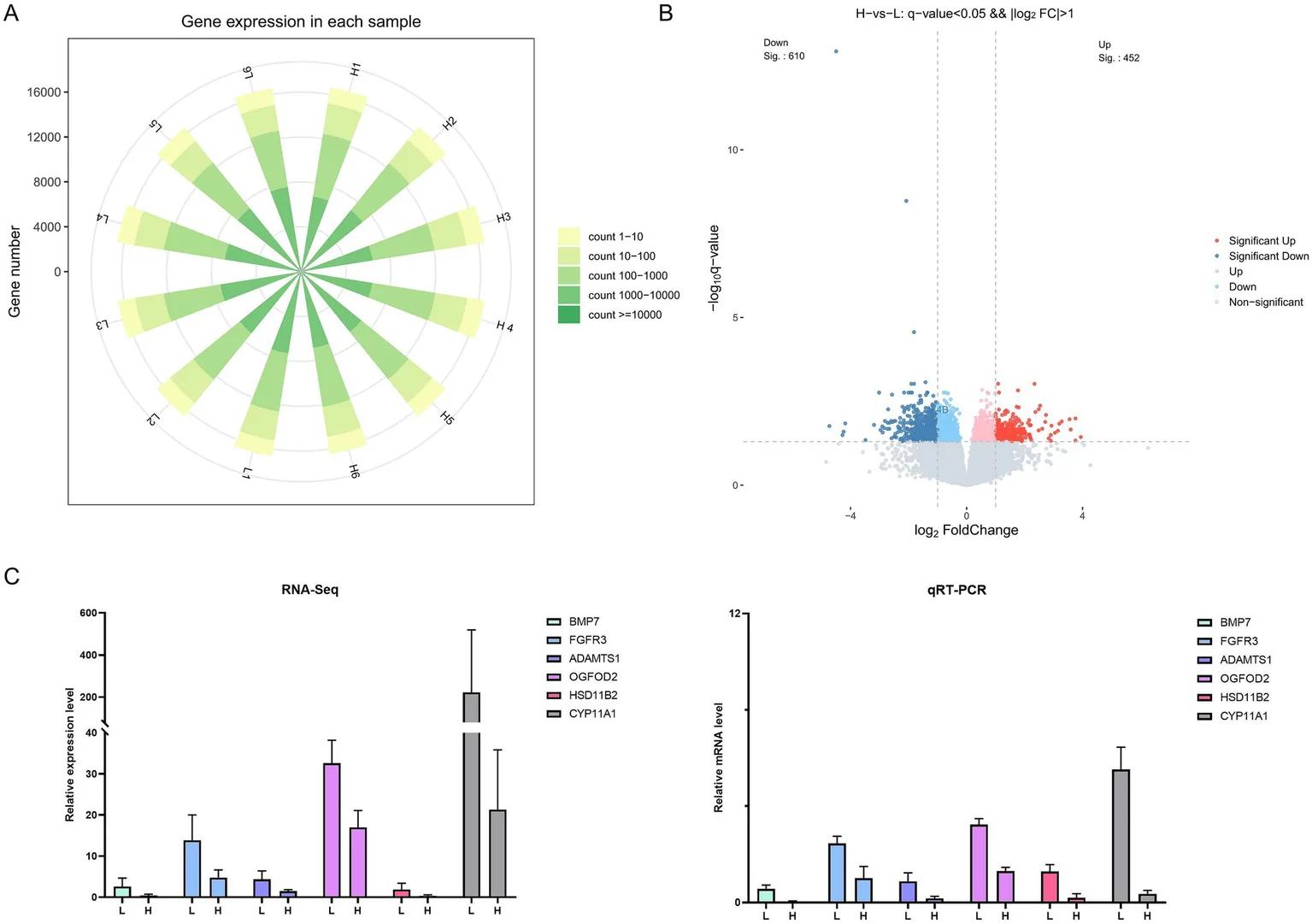
**(A)** Statistical chart of detected gene counts from RNA-Seq. Each sector represents the gene count of an individual sample, with different colors indicating gene count intervals. **(B)** Volcano plot depicting DEGs identified by RNA-Seq. Gray dots represent non-significant DEGs, while red and blue dots indicate significantly up- and down-regulated DEGs, respectively. **(C)** qRT-PCR validation plot for selected DEGs. The left panel shows RNA-Seq results, and the right panel shows qRT-PCR results for the same DEGs.

To investigate the molecular basis underlying differences in sperm quality, the testicular transcriptomes of ganders between L and H groups were analyzed. As shown in Supplementary Table 2, 48,345,968 ~ 49,809,212 raw reads were obtained from these 12 samples, and an average of 47,802,565 clean reads were obtained from each sample after a stringent quality control. The uniquely mapped rates for all samples ranged from 88.15% to 91.05%. Collectively, these data attest to the robust quality and biological reproducibility, enabling its use in subsequent bio-informatics analyses. Genes with zero expression counts across all 12 samples were filtered out based on the database of known reference gene sequences and annotation files. The final number of detected genes ultimately detected in the samples ranged from 21,427 to 21,929 (Figure 4A). Through comparative analysis, a total of 1,062 genes (452 upregulated and 610 downregulated) were differentially expressed between H and L groups, as shown in Figure 4B.

### 3.4 Validation of RNA-Seq results using qRT-PCR

qRT-PCR was applied to validate the transcriptional alterations of DEGs identified through RNA sequencing. Six DEGs were chosen at random for experimental detection. The expression tendencies of these genes measured by qRT-PCR matched well with the transcriptome results, proving the good credibility and precision of our sequencing data (Figure 4C).

### 3.5 Gene ontology (GO) and KEGG analyses

**Figure 5:**
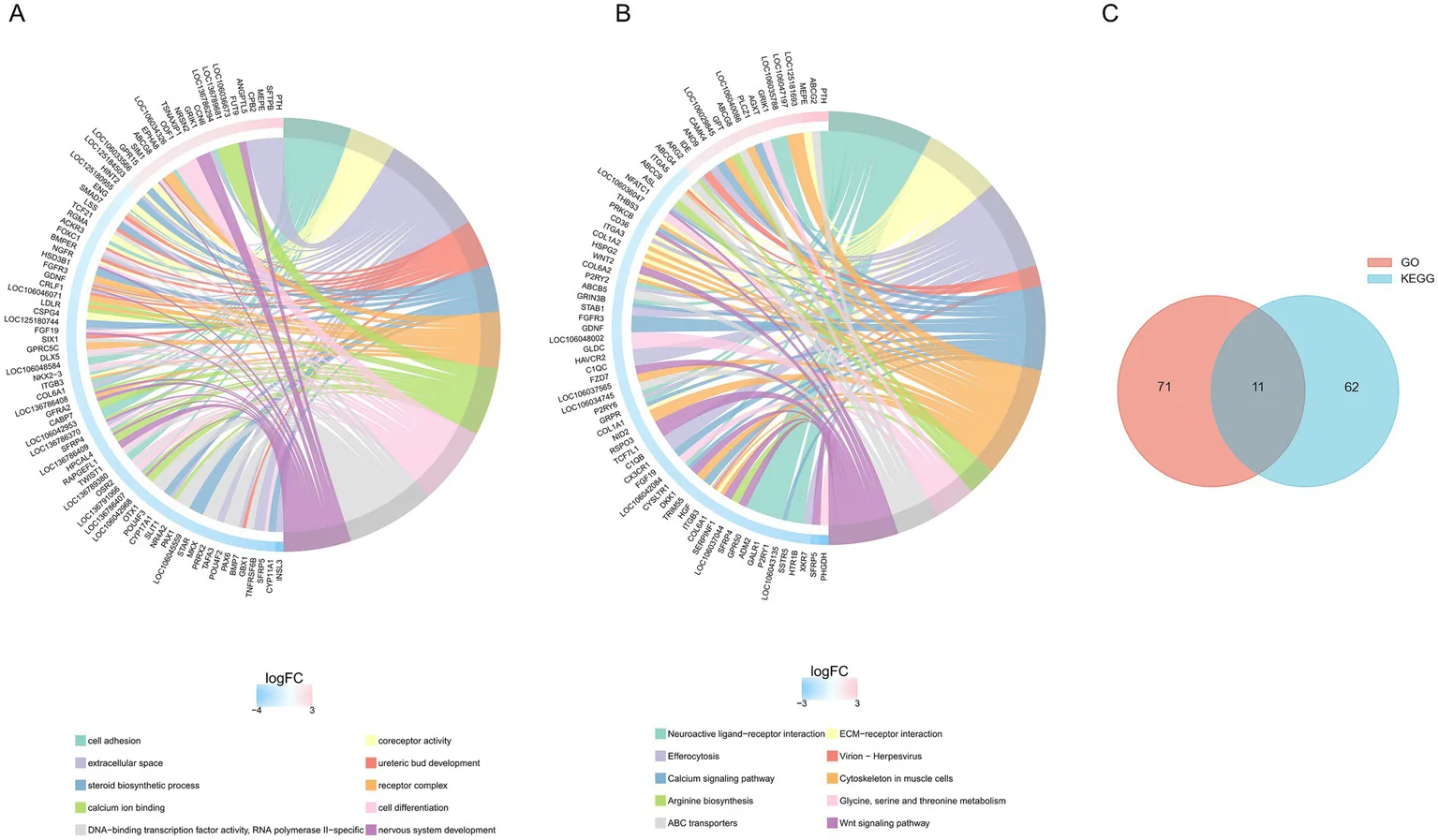
Integrative enrichment analysis of DEGs. **(A)** Circos plot of top 10 significantly enriched GO terms, the left side displays the top 10 genes with the highest |*logFC*| in each category, the inner heatmap represents the *logFC* values of the corresponding genes, and the right side reflects the composition of GO term. **(B)** Circos plot of top 10 KEGG significantly enriched pathways, the left side displays the top 10 genes with the highest |*logFC*| in each category, the inner heatmap represents the *logFC* values of the corresponding genes, and the right side reflects the composition of each signaling pathway. **(C)** Venn diagram of DEGs identified by the top 10 GO and top 10 KEGG enrichment analysis.

To explore the potential biological functions of DEGs associated with testicular physiological function, we performed GO enrichment analysis. The top 10 significantly enriched GO terms derived from the H vs. L comparison were visualized in Figure 5A, which predominantly included cell adhesion, cell differentiation, steroid biosynthetic process, calcium ion binding, and so on. In total, 82 DEGs were successfully annotated to these top enriched GO terms, covering multiple core biological processes closely related to testicular development and spermatogenesis. Furthermore, KEGG pathway enrichment analysis was conducted to characterize the key signaling pathways and interactive networks underlying the differential gene expression. The top 10 most enriched KEGG pathways for the H vs. L comparison are displayed in Figure 5B, with representative pathways including the ECM-receptor interaction, calcium signaling pathway, Wnt signaling pathway, and so on. Collectively, 73 DEGs were annotated to these pivotal pathways, indicating that the dysregulation of these signaling cascades may be the crucial molecular basis for the differences in testicular biological function between the two groups. Venn analysis was performed among the enriched DEGs in top 10 GO terms and top 10 KEGG pathways. The Venn plot (Figure 5C) illustrated the overlap between DEGs from GO and KEGG enrichment, with a total 144 DEGs obtained. There were 82 DEGs associated with the top 10 GO terms, 73 DEGs related to the top 10 KEGG pathways, 11 genes were overlapping in both sets: *SFRP5*, *FGFR3*, *GDNF*, *COL6A1*, *PTH*, *MEPE*, *SFRP4*, *ITGB3*, *GRIK1*, *FGF19*, and *ABCG8*.

### 3.6 Network analysis of gander testicular and epididymal DEGs

**Figure 6:**
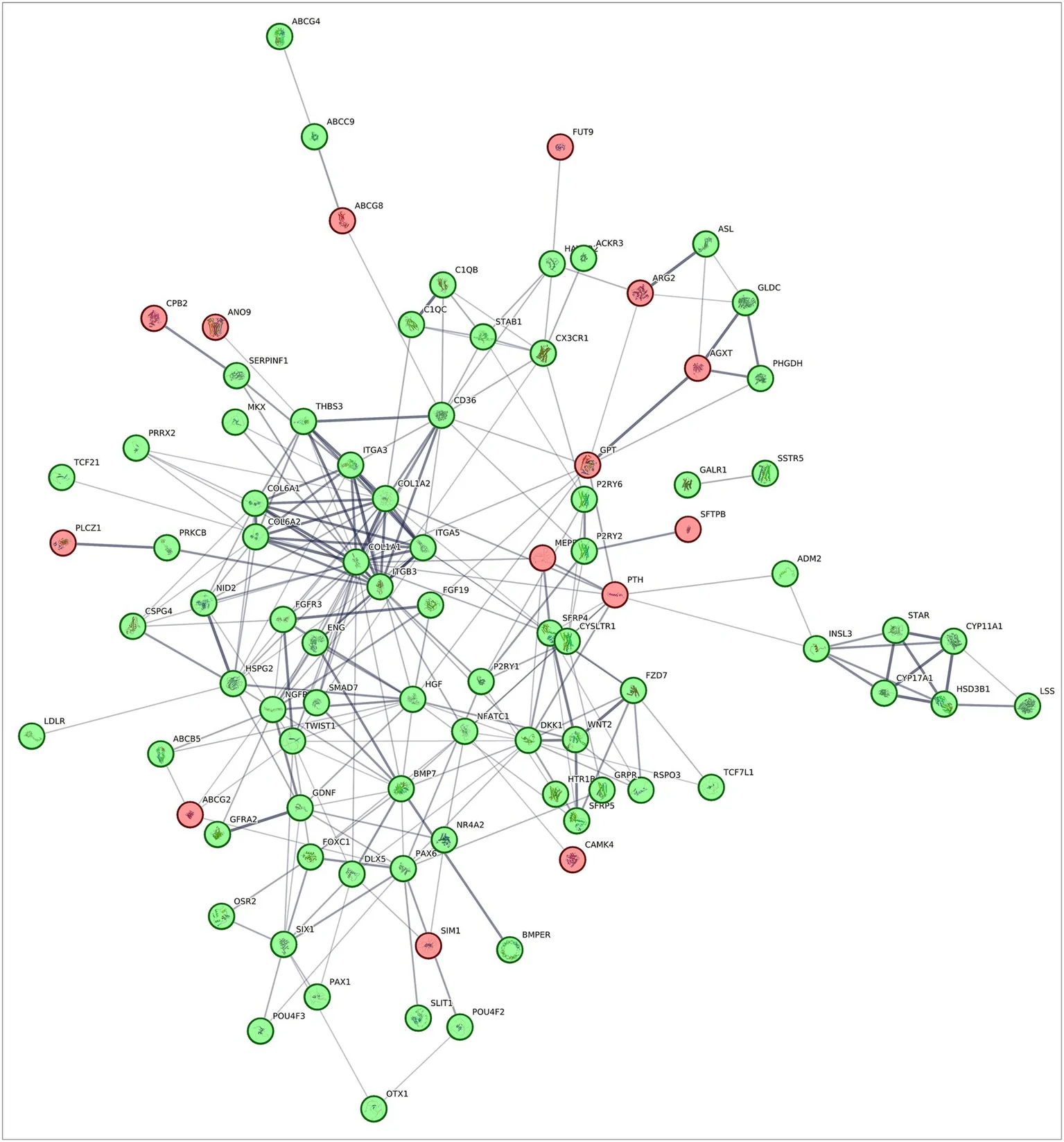
PPI network analysis of DEGs enriched in top 10 GO and top 10 KEGG related to the gander semen quality that were identified by RNA-Seq between L and H groups. Green nodes represent down-regulated DEGs, and red nodes represent up-regulated DEGs.

To screen hub genes and key pathways affecting semen quality, a PPI network was constructed by using the 144 DEGs obtained from Venn analysis. The network consisted of 65 nodes and 244 edges (Figure 6). Comparative analysis showed that merely 14 DEGs were significantly upregulated in the H vs. L comparison group, whereas the vast majority were downregulated. Using Cytoscape to calculate node degree, we acquired 11 hub genes (Table 2) with the highest connectivity: *COL1A1*, *HGF*, *COL1A2*, *ITGA5*, *NGFR*, *TWIST1*, *COL6A2*, *DKK1*, *BMP7*, *WNT2* and *CYP11A1*. KEGG pathway enrichment indicated that these hub genes were primarily annotated to ECM-receptor interaction, Cytoskeleton in muscle cells and Wnt signaling pathway. In addition, *COL6A1* was a common gene between the top GO and KEGG enriched DEGs, and was identified as a hub gene in the PPI network.

**Table 2:** The key genes identified in PPI network constructed with the testicular DEGs.

Gene	Gene name	Degree	KEGG pathways/GO terms	Regulation
*COL1A1*	Collagen type I alpha 1 chain	25	ECM-receptor interaction, Cytoskeleton in muscle cells/extracellular region	Down
*HGF*	Hepatocyte growth factor	13	Calcium signaling pathway/extracellular space	Down
*COL1A2*	Collagen type I alpha 2 chain	12	ECM-receptor interaction, Cytoskeleton in muscle cells/ extracellular space	Down
*ITGA5*	Integrin subunit alpha 5	10	ECM-receptor interaction, Virion-Herpesvirus, Cytoskeleton in muscle cells/cell surface	Down
*NGFR*	Nerve growth factor receptor	9	Virion-Ebolavirus/cell differentiation	Down
*TWIST1*	Twist family bHLH transcription factor 1	9	Wnt signaling pathway/cell differentiation, DNA-binding transcription factor activity, RNA polymerase II-specific	Down
*COL6A2*	Collagen type VI alpha 2 chain	7	ECM-receptor interaction, Cytoskeleton in muscle cells/ cell adhesion, extracellular space	Down
*DKK1*	dickkopf Wnt signaling pathway inhibitor 1	7	Wnt signaling pathway/positive regulation of gene expression, extracellular space, extracellular region	Down
*BMP7*	Bone morphogenetic protein 7	6	TGF-beta signaling pathway/ureteric bud development, positive regulation of gene expression, extracellular space, extracellular region	Down
*WNT2*	Wnt family member 2	5	Wnt signaling pathway/extracellular region	Down
*CYP11A1*	Cytochrome P450 family 11 subfamily A member 1	5	Steroid hormone biosynthesis/steroid biosynthetic process	Down
*COL6A1*	Collagen Type VI Alpha 1 Chain	5	ECM-receptor interaction, Cytoskeleton in muscle cells/cell adhesion, extracellular space	Down

## 4 Discussion

Reproductive performance constitutes a primary economic driver in poultry production, and its improvement has consistently shaped research priorities in avian husbandry (25). In breeding ganders, semen quality serves as a robust predictor of fertility, directly influencing not only fertilization success but also offspring growth, survival, and long-term productivity (11, 26). Consequently, systematic semen quality assessment is indispensable for the selection of elite breeding individuals, forming the cornerstone of sustainable reproductive efficiency and output in commercial goose flocks. Nevertheless, local goose breeds in numerous countries—China included—frequently exhibit compromised semen quality, manifesting as reduced sperm motility, elevated morphological abnormalities, and decreased sperm concentration (27). These deficiencies represent a major bottleneck for the goose industry, as they directly impair fertilization rates, hatchability, and overall economic viability. Given the steadily growing global demand for high-quality goose products, the development of scalable and practical interventions to enhance gander reproductive efficiency has become an urgent research imperative. In this study, we performed a comparative analysis of semen quality parameters, testicular and external reproductive organ histomorphology, and testicular transcriptomic profiles between ganders with high and low semen quality to reveal the molecular basis of semen quality in ganders.

Our study reveals substantial heterogeneity in semen quality among male Zhedong geese, a Chinese local breed, with SQF ranging widely from 0.37 to 63.14 (*n* = 6). To investigate the biological basis of this variability, we categorized geese into high sperm quality (G) and low sperm quality (L) groups using the upper quartile (27.09) and lower quartile (4.35) of SQF values as cut-offs, respectively. Comparative analysis revealed that group H had significantly higher ejaculate volume, sperm density, sperm motility, and SQF than group L (*p* < 0.01). Additionally, H group had significantly longer helical, natural, and stretched lengths of the external genitalia compared to group L (*p* < 0.05). Testicular weight, volume, and organ index were also significantly higher in H group than in L group (*p* < 0.05), suggesting a positive correlation between reproductive organ development and semen quality. Histological observations supported these findings: H group had significantly increased SET and STD compared to group L (*p* < 0.01). Similar differences have been reported in other poultry species with varying semen quality (18, 28). Testicular development is known to directly influence sexual behavior, semen quality, and external genitalia development in male poultry (29, 30). Seminiferous tubule diameter correlates positively with spermatogenic capacity (31), and spermatogenic cell development is a key determinant of semen quality and fertilization ability (26). Seminiferous epithelium thickness is also an important indicator of male reproductive function (32). Collectively, these results suggest that testicular morphological and histological differences may be the primary cause of variations in external genitalia development and semen quality in male geese.

Through comparative transcriptomic analysis of testicular tissues, we identified a total of 452 upregulated and 610 downregulated DEGs between H and L groups. Validation using qRT-PCR confirmed that the expression trends of these DEGs were consistent with the RNA-seq results, indicating that the RNA-seq data were reliable and suitable for subsequent in-depth analysis. GO enrichment analysis of the top 10 most significantly enriched terms revealed that these DEGs were widely distributed across biological process, cellular component, and molecular function categories. Notably, terms related to cell adhesion (33), steroid biosynthetic processes (34), and calcium ion binding (35) were highlighted as potentially important for association with gander sperm quality. Furthermore, KEGG pathway enrichment analysis of the top 10 most significantly enriched pathways identified significant enrichment in ECM-receptor interaction (36), calcium signaling pathway (37), and Wnt signaling pathway (38). These pathways have been extensively reported to play critical roles in regulating sperm quality and testicular development across various species, including geese. To narrow down the candidate genes with potential functional relevance, we intersected the genes from the top 10 GO terms and top 10 KEGG pathways, reducing the initial set of 1,062 DEGs to 166 candidate genes by Venn analysis. Among these, 11 genes were co-enriched in both GO and KEGG analyses, suggesting they may serve as candidate hub genes in regulating gander sperm quality.

To further identify hub genes potentially associated with semen quality in ganders, we performed protein–protein interaction (PPI) network analysis and screened 14 candidate genes with the highest node degrees using Cytoscape. Notably, all 14 candidate genes were down-regulated in the H group in the present study. A similar expression pattern was also observed in a previous goose study by Hu et al. (18), in which they classified individuals based on sperm motility, whereas our study employed a composite SQF as the core grouping indicator. SQF is a comprehensive parameter that integrates multiple semen quality dimensions, thereby providing a more holistic reflection of an individual’s overall semen quality level. Similarly, their PPI network analysis of key genes revealed that most testicular hub DEGs were down-regulated between the two groups, further supporting the notion that down-regulation of specific testicular genes may be associated with enhanced semen quality. Furthermore, in the testis, Hu et al. (18) identified 5 key DEGs (*PLCB1*, *PLCB2*, *WNT11*, *WNT4*, and *LRP6*), all of which were significantly enriched in the Wnt signaling pathway. In contrast, our data analysis highlighted 3 key DEGs (*TWIST1*, *DKK1*, and *WNT2*) that were also significantly enriched in the Wnt signaling pathway. Despite the differences in the specific hub genes identified, both studies consistently suggest the Wnt signaling pathway as a potential key pathway involved in testicular function and sperm motility in ganders, underscoring its conserved and potentially important role in avian male reproduction. Among these 14 candidate genes, we are particularly interested in three of these candidate genes (*CYP11A1*, *NGFR*, and *COL6A1*) due to the above gene function predictions and their documented roles in testicular function across multiple species.

*CYP11A1* (cytochrome P450 family 11 subfamily A polypeptide 1), a key mitochondrial enzyme initiating steroidogenesis by converting cholesterol to pregnenolone, serves as the rate-limiting step in testosterone biosynthesis. Studies in chickens have demonstrated that *CYP11A1* is expressed in ovarian granulosa cells and regulates steroid hormone production (39), while in mouse Leydig cells, it plays an essential role in testosterone synthesis and spermatogenesis regulation (40). Notably, our network analysis identified *CYP11A1* as a central node in the Steroid hormone biosynthesis pathway, suggesting its potential function in regulating gander testicular development and semen quality through testosterone production. However, Liu et al. reported (41) that *CYP11A1* was up-regulated in the testicular transcriptome of high-sperm-motility Landes ganders, whereas in the present study, *CYP11A1* was down-regulated in the H group. This discrepancy may be attributable to several factors. First, developmental stage and genetic background differ between the two studies: the previous study used 2-year-old Landes geese, while our study employed approximately 30-week-old Zhedong White ganders. Second, whole-testis RNA-seq inherently confounds cell-type composition with gene expression. *CYP11A1* is primarily expressed in Leydig cells within the interstitium (40), whereas germ cells constitute the bulk of testicular volume. Histological observations revealed that the H group had larger seminiferous tubules containing abundant germ cells and relatively reduced interstitial space, whereas the L group displayed numerous smaller tubules with increased interstitial tissue. This compositional shift would dilute the relative contribution of Leydig cells to the total RNA pool, potentially leading to reduced *CYP11A1* transcript abundance at the whole-tissue level without reflecting single-cell transcriptional downregulation. Third, negative feedback via the hypothalamic–pituitary-gonadal axis likely plays a pivotal role. Previous studies have shown that serum testosterone levels are elevated in high semen quality groups (17), which would consequently inhibit expression of steroidogenic genes in Leydig cells, such as *CYP11A1*. Of note, *STAR* (Steroidogenic Acute Regulatory Protein), which acts upstream of *CYP11A1* by transporting cholesterol to the inner mitochondrial membrane for *CYP11A1*-catalyzed conversion to pregnenolone (42), was also down-regulated in the H group in our study. In contrast, Liu et al. (41) reported no significant difference in *STAR* expression between the high- and low-motility groups in Landes ganders, further illustrating that the regulatory pattern of steroidogenic genes may differ between breeds and developmental stages. Additionally, through PPI network analysis, we identified a regulatory axis involving *CYP11A1* (−)/*STAR* (−)/*INSL3* (−)/*PTH* (+), in which *PTH* (Parathyroid Hormone) was significantly up-regulated. *PTH* is a polypeptide hormone secreted by parathyroid chief cells and has been reported to effectively increase serum testosterone levels and promote stem Leydig cell differentiation in mice (43). The concurrent down-regulation of *CYP11A1* in our study further supports the notion that reduced steroidogenic gene expression in the H group may be attributed to compositional shifts and negative feedback regulation, rather than a simple gene-specific response. Nevertheless, the precise regulatory mechanisms linking *CYP11A1* down-regulation to semen quality in Zhedong White ganders warrant further investigation.

*NGFR* (nerve growth factor receptor), a transmembrane receptor highly expressed in male germ cells, has been implicated in multiple aspects of testicular function. In mice, *NGFR* expression shifts from embryonic mesenchymal cells to the seminiferous tubule periphery postnatally, where it regulates meiosis initiation and spermatogonial stem cell maintenance (44). Immunohistochemical studies in rats (45) have localized *NGFR* to Sertoli cells, Leydig cells, pachytene spermatocytes, and elongating spermatids, indicating its multifaceted role in testicular cell communication and spermatogenesis progression. Our PPI analysis revealed *NGFR*’s interaction with several spermatogenesis-related genes, suggesting it may act as a candidate signaling hub potentially linking neurotrophic factors with testicular development in ganders. *COL6A1* (collagen type VI alpha 1 chain), encoding the alpha-1 chain of type VI collagen, represents an intriguing candidate with emerging links to male fertility. Transcriptomic analysis in buffalo testes and spermatozoa detected elevated *COL6A1* expression, leading to the hypothesis that it contributes to sperm motility and fertilization potential through extracellular matrix reorganization (46). In our study, *COL6A1* was identified as a common gene between the top GO and KEGG enriched DEGs and was also recognized as a hub gene in the PPI network. Its network positioning at the intersection of cell adhesion (47) and extracellular space further highlights its potential as candidate gene associated with semen quality in ganders. Despite the insights, several limitations should be acknowledged, including the relatively small sample size (six ganders per group) and the transcriptome-only nature of our analysis, which may not fully capture post-transcriptional or translational regulatory events. Future integration of proteomic or metabolomic data, along with functional validation of the identified hub genes through function experiments, will be essential to further elucidate the molecular mechanisms governing semen quality and to establish a solid foundation for genetic improvement strategies in goose breeding.

## 5 Conclusion

In conclusion, this study demonstrates a strong positive correlation between semen quality, testicular morphology, and external genitalia morphology in male geese, suggesting structural-functional integration underlying reproductive performance. Comparative transcriptomic analysis identified candidate key genes and signaling pathways distinguishing high and low semen quality groups. These findings advance understanding of male goose reproductive physiology, reveal conserved molecular mechanisms potentially involved in semen quality, and offer potential molecular markers for selective breeding programs to improve reproductive efficiency in commercial goose production.

## Data Availability

The data presented in the study are deposited in the National Genomics Data Center repository, accession number GSA: CRA030664 (https://ngdc.cncb.ac.cn/gsa/s/fIl9N5BK).
