# Supplementary material for: Comparative testicular transcriptomic analysis reveals hub genes and pathways regulating semen quality in Zhedong white ganders (Anser cygnoides)
Source: Front Vet Sci. 2026 Aug 12;13:1906421. doi: 10.3389/fvets.2026.1906421 (PMC13506476; doi:10.3389/fvets.2026.1906421)
Supplement: Supplementary file 1 [file Table_1.DOCX]

**Table S1 Comparison results of clean data and reference genome from RNA-Seq**

| Sample | Total reads | Total mapped reads | Multiple mapped | Uniquely mapped |
| --- | --- | --- | --- | --- |
| D1 | 49809212 | 48736949(97.85%) | 4396510(8.83%) | 44340439(89.02%) |
| D2 | 49096928 | 48079811(97.93%) | 3687876(7.51%) | 44391935(90.42%) |
| D3 | 48788940 | 47783491(97.94%) | 4775602(9.79%) | 43007889(88.15%) |
| D4 | 48614896 | 47579176(97.87%) | 3675900(7.56%) | 43903276(90.31%) |
| D5 | 48460832 | 47314562(97.63%) | 4449087(9.18%) | 42865475(88.45%) |
| D6 | 48540944 | 47481135(97.82%) | 4395859(9.06%) | 43085276(88.76%) |
| G1 | 48378978 | 47274078(97.72%) | 3595365(7.43%) | 43678713(90.28%) |
| G2 | 49345968 | 48248939(97.78%) | 3753949(7.61%) | 44494990(90.17%) |
| G3 | 48776902 | 47716618(97.83%) | 3303171(6.77%) | 44413447(91.05%) |
| G4 | 49331066 | 48233988(97.78%) | 3512115(7.12%) | 44721873(90.66%) |
| G5 | 48359298 | 47312574(97.84%) | 3963467(8.20%) | 43349107(89.64%) |
| G6 | 48794318 | 47869458(98.10%) | 3582012(7.34%) | 44287446(90.76%) |
