# Supplementary material for: Comparative testicular transcriptomic analysis reveals hub genes and pathways regulating semen quality in Zhedong white ganders (Anser cygnoides)
Source: Front Vet Sci. 2026 Aug 12;13:1906421. doi: 10.3389/fvets.2026.1906421 (PMC13506476; doi:10.3389/fvets.2026.1906421)
Supplement: Supplementary file 2 [file Table_2.DOCX]

**Table S2 Primer information for qRT-PCR**

| Gene | Sequence (5′-3′) | Tm (°C) | Product length (bp) |
| --- | --- | --- | --- |
| *BMP7* | F: CTGCTGTGCTCCTACCCAACTC | 60 | 101 |
|  | R: ATGCTCGTACCACCATGTTCCTG |  |  |
| *FGFR3* | F: CACGCTCTCGCCTGACCTTG | 60 | 89 |
|  | R: GTCCTTATCAATCCCAATCGCTTCTG |  |  |
| *ADAMTS1* | F: CACTACCTCCTGACGCTGTTCTC | 58 | 110 |
|  | R: CGCTCCTCGTAGATGACCATGATC |  |  |
| *OGFOD2* | F: GCTACACCGAGTCCTTCTCAGATAAG | 60 | 108 |
|  | R: TGTCCGACTGCTCAAAGTTCTCC |  |  |
| *HSD11B2* | F: CCGCTACCTGCCCTATTTCGTC | 60 | 108 |
|  | R: GCCTCCTTCGCCTCAGTGTG |  |  |
| *CYP11A1* | F: GACCGCCTGACCCTGAACAAG | 58 | 123 |
|  | R: GCCGCTCTTCTGGACCTGTG |  |  |
| *GADPH* | F: GTGAAAGTCGGAGTCAACGG | 60 | 79 |
|  | R: CTTGGACTTTGCCAGACAGGA |  |  |
| *β-ACTIN* | F: TATTGCTGCGCTCGTTGTTG | 60 | 93 |
|  | R:ACGATGGATGGGAAGACAGC |  |  |
